# Comparison of the analytical performance between the Oncomine Dx Target Test and a conventional single gene test for epidermal growth factor receptor mutation in non‐small cell lung cancer

**DOI:** 10.1111/1759-7714.13767

**Published:** 2020-12-12

**Authors:** Tadashi Sakaguchi, Akemi Iketani, Kazuki Furuhashi, Yuki Nakamura, Yuta Suzuki, Kentaro Ito, Kentaro Fujiwara, Yoichi Nishii, Koji Katsuta, Osamu Taguchi, Osamu Hataji

**Affiliations:** ^1^ Respiratory Center Matsusaka Municipal Hospital Matsusaka Japan; ^2^ Pathology Department Matsusaka Municipal Hospital Matsusaka Japan

**Keywords:** *EGFR* mutations, next‐generation sequencing, non‐small cell lung cancer, Oncomine Dx Target Test, PNA‐LNA PCR clamp

## Abstract

**Background:**

Next‐generation sequencing (NGS) has been implemented in clinical oncology to analyze multiple genes and to guide targeted therapy; however, little is known about the performance of the Oncomine Dx Target Test compared with conventional single gene tests for detecting *EGFR* mutations. The objective of this study was to evaluate the performance of the Oncomine Dx Target Test compared with a PNA‐LNA PCR clamp test to detect *EGFR* mutations.

**Methods:**

We retrospectively reviewed consecutive patients with non‐small cell lung cancer (NSCLC) from whom FFPE samples were simultaneously submitted for the Oncomine Dx Target Test, and a PNA‐LNA PCR clamp test using the same specimen. We subsequently compared the analysis success rates and detection rates between the two tests.

**Results:**

A total of 116 samples were identified. The success rates and detection rates of *EGFR* mutations in the total number of samples were 90% and 28%, respectively for the Oncomine Dx Target Test, and 100% and 35% for the PNA‐LNA PCR clamp test. The Oncomine Dx Target Test was unable to analyze three samples (2%) due to the samples not passing the nucleic acid concentration threshold, and nine (8%) samples had invalid results. The exon 19 deletion was not detected by the Oncomine Dx Target Test in four cases (4%).

**Conclusions:**

The analytical performance of the Oncomine Dx Target Test analysis for *EGFR* mutations may not be comparable with conventional single gene tests due to both invalid and false‐negative results.

**Key points:**

**Significant findings of the study**

- The success rate of the Oncomine Dx Target Test was significantly lower than the PNA‐LNA PCR clamp test.
- Among the samples successfully analyzed, four exon 19 deletions were not detected by the Oncomine Dx Target Test.

**What this study adds**

- The analytical performance of the Oncomine Dx Target Test may not be comparable with conventional single gene tests.
- We should revise the sampling procedures, and review the sample quality assessment methods, to improve the analytical performance.

## Graphical abstract

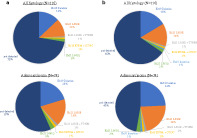

There have been few studies on the performance of the Oncomine Dx Target Test compared with a PNA‐LNA PCR clamp test. Here, we reviewed 116 samples and compared the success and detection rates of *EGFR* mutations. The rates of the Oncomine Dx Target Test were numerically lower than the PNA‐LNA PCR clamp test, and analytical performance between the two tests may therefore not be comparable.

## Introduction

Targeted therapies for non‐small cell lung cancer (NSCLC) patients harboring driver oncogene alterations have been proven to have promising antitumor activities, and are generally recommended in the clinical guidelines.^1^, ^2^, ^3^ Epidermal growth factor receptor‐tyrosine kinase inhibitors (EGFR‐TKIs) play an important role in effective therapy, especially for NSCLC patients in East Asia.^4^

Conventional single‐gene tests, such as the cobas *EGFR* assay, the therascreen *EGFR* assay as an in vitro diagnostic (IVD) test, and the peptide nucleic acid‐locked nucleic acid polymerase chain reaction (PNA‐LNA PCR) clamp assay as a laboratory‐developed test (LDT) for *EGFR* mutations, have been conducted to select suitable patients responsive to EGFR‐TKI.^5^, ^6^, ^7^ Recently, as more gene targets have been identified and recognized in clinical settings for NSCLC, more biomarker tests have been required to make appropriate treatment decisions. As the number of single‐gene tests has increased, tissue consumption for analysis of multiple single‐gene tests has also increased, and the completion rates of the ordered tests has decreased.^8^

Next‐generation sequencing (NGS) can detect multiple gene variants simultaneously enabling comprehensive genetic testing. The Oncomine Dx Target Test (Ion Torrent PGM Dx Sequencer; Thermo Fisher Scientific) is an NGS panel for NSCLC, approved by the US Food and Drug Administration in June 2017.^9^ It is a qualitative, in vitro diagnostic test that uses high‐throughput parallel sequencing technology to detect sequence variations in 46 genes on DNA and RNA isolated from formalin‐fixed and paraffin‐embedded (FFPE) specimens using the Ion PGM Dx System. In February 2019, the test was approved for use in Japan as a companion diagnostic for targeted therapies on four driver mutations; *EGFR*, anaplastic lymphoma kinase (*ALK*), ROS proto‐oncogene 1, receptor tyrosine kinase (*ROS1*), and B‐Raf proto‐oncogene, serine/threonine kinase (*BRAF*) (p.V600E).

The Oncomine Dx Target Test is expected to analyze multiple gene targets reliably and simultaneously; however, the performance of the Oncomine Dx Target Test compared with conventional single gene tests for detecting *EGFR* mutations has not previously been fully evaluated. Therefore, in this study, we retrospectively evaluated the performance of the Oncomine Dx Target Test compared with the PNA‐LNA PCR clamp test, in detecting *EGFR* mutations in patients with NSCLC.

## Methods

### Patient selection

This retrospective study was conducted at Matsusaka Municipal Hospital, Japan. We reviewed the electronic data from consecutive NSCLC patients whose FFPE samples were simultaneously submitted for an Oncomine Dx Target Test, and a PNA‐LNA PCR clamp test, using the same specimen, from August 2019 to February 2020. Samples collected in other hospitals, and archived samples were excluded. Clinical data assessments included patient characteristics, sampling methods, staging, histology, pathological findings, and the results of genetic tests. This study was approved by the institutional review board of Matsusaka Municipal Hospital (Approval date: 20 April 2020; IRB number J‐76‐200 410‐5‐2). Informed consent was obtained by the opt‐out method.

### Sample processing and genetic tests

Small tissue samples collected by endobronchial biopsy/transbronchial biopsy (EBB/TBB), computed tomography‐guided percutaneous needle biopsy (CTNB) and fine needle aspiration (FNA) were immediately placed in 10% neutral buffered formalin (NBF) and fixed over 12 to 18 hours at room temperature. In cases of surgical lung resection, the samples from limited resections, including lung segmentectomy and wedge resections, were immediately placed in 10% NBF after sampling for intraoperative rapid diagnosis (IRD) for 24 to 48 hours at room temperature. The specimens from lung lobectomies performed up to January 2020 were stored in a refrigerator at 4°C for less than three hours, after sampling for IRD, then placed in 10% NBF for 24 to 48 hours at room temperature. Meanwhile, using samples from lung lobectomies performed after January 2020, a tissue sample of the correct size (10 mm × 10 mm), and enriched in tumor cells was prepared for the Oncomine Dx Target Test concurrently when sampling for IRD, and placed in 10% NBF immediately after sampling. Formalin‐fixed tissues underwent serial processing and were then embedded in paraffin to create FFPE blocks. The amount of tumor cells, and tumor content of the sample stained with hematoxylin and eosin, were evaluated by skilled cytopathologists. Trimming, which eliminates samples containing little or no tumor cells in small biopsy samples. was performed, but macro‐ and microdissection were not performed in our institution. If the tumor content was <20% after trimming of small biopsy samples, or the amount of tumor cells was insufficient, the sample was not submitted for the Oncomine Dx Target Test. For the Oncomine Dx Target Test, 10 to 15 slide‐mounted 5 μm sections of small biopsy samples and 5 to 10 slide‐mounted 5 μm sections of surgical resection samples were submitted to LSI Medience Laboratories (Tokyo, Japan). For the PNA‐LNA PCR clamp test, five slides of 5 μm sections from small biopsy samples and surgical resection samples were submitted. LSI Medience Laboratories performed the Oncomine Dx Target Tests based on Thermo Fisher's Ion AmpliSeq technology, and the PNA‐LNA PCR clamp tests were performed using the PNA‐LNA PCR clamp assay.^7^, ^9^ The Oncomine Dx Target Test panel included 46 genes (Table SS1).

### Specific *EGFR* mutations detectable by each test

Specific *EGFR* mutations detectable by the Oncomine Dx Target Test were identified in exon 18 (p.G709X and p.G719A/C/S/D), exon 19 (deletion), exon 20 (p.S768I and p.T790M), and exon 21 (p.L858M, p.L858R and p.L861Q/R) and other mutations in exon 18 to 21. Exon 20 insertion has been included in Oncomine Dx Target Test reports since May 2020 in Japan. Specific *EGFR* mutations detectable by PNA‐LNA PCR clamp test were identified in exon 18 (p.G709X and p.G719A/C/S), exon 19 (deletion and insertion), exon 20 (p.S768I, p.T790M and insertion), and exon 21 (p.L833X, p.L858R and p.L861Q) and other mutations in exon 18 to 21 (p.V769M, p.V834L, p.K860I, etc.). p.S768I, exon 20 insertion and p.L833X have been included in PNA‐LNA PCR clamp test reports since October 2019.

### Outcomes

We evaluated the success rate and detection rate of *EGFR* mutations for the Oncomine Dx Target Test compared with those of the PNA‐LNA PCR clamp test. The analysis results were regarded as successful if all the results in the following *EGFR* mutations reported for each time period were completely available; exon 18 (p.G719A/C/S), exon 19 (deletion), exon 20 (p.S768I, p.T790M, insertion), and exon 21 (p.L858R and p.L861Q), which are the mutations considered required for detection due to clinical implications by the Japanese Lung Cancer Society. Meanwhile, the analysis results were regarded as unsuccessful if the sample did not pass the nucleic acid concentration threshold, or if one or more *EGFR* sequence results mentioned above were invalid due to a failure to meet the DNA sample quality control (QC) metrics, or no call. The detection rates of *EGFR* mutation were calculated as the rates of samples with detected *EGFR* mutations in all samples, or samples diagnosed with adenocarcinoma. We also evaluated concordance rate, and the clinical settings of patients with the discordant results between the two tests. The concordance rate was calculated as follows: concordance rate = Nc/Nt, where Nc was the number of samples showing the concordant results, and Nt was the total number of successfully analyzed samples.

### Statistical analysis

Statistical analyses were performed using Pearson's Chi‐squared test for comparison of analysis success rates and detection rates. *P*‐values less than 0.05 indicated statistical significance. Statistical analyses were performed using SPSS software, version 26.0 (SPSS Inc., Chicago, USA).

## Results

### Sample characteristics

A total of 116 samples were identified for comparison analysis. The sample characteristics for the analysis are shown in Table 1. The main sampling methods were EBB/TBB (60%), surgical resection (21%), and CTNB (16%). More than half of the tumor histology was adenocarcinoma (64%), followed by squamous cell carcinoma (29%).

**Table 1:** Sample characteristics

	Total samples	
	*N* = 116	(%)
Sampling method		
EBB/TBB	69	60%
Surgical resection	24	21%
CTNB	19	16%
Others	4	3%
Histology		
ADC	74	64%
Sq	34	29%
Non‐Sq Non‐ADC	7	6%
NSCC NOS	1	1%

ADC, adenocarcinoma; CTNB, computed tomography‐guided needle aspiration; EBB, endobronchial biopsy; NSCC NOS, non‐small cell carcinoma, not otherwise specified; Sq, squamous cell carcinoma; TBB, transbronchial biopsy.

### Success and detection rates for *EGFR* mutations for each test

The success rates for the Oncomine Dx Target Test and PNA‐LNA PCR clamp test are shown in Table 2. Although the success rate of the Oncomine Dx Target Test was 90%, the success rate of the PNA‐LNA PCR clamp test was 100% (*P* < 0.01). *EGFR* mutation results from the Oncomine Dx Target Test could not be assessed in three samples (2%) due to a failure to pass the nucleic acid concentration threshold. Nine samples returned invalid results; seven samples (6%) generated invalid results due to a failure to meet the DNA sample QC metrics, or no call for all *EGFR* mutations specified above, and two samples (2%) generated invalid results for the subset of the mutations due to no call.

**Table 2:** Analysis success rates of Oncomine Dx Target Test and PNA‐LNA PCR clamp test

	Total samples *N* = 116			
	Oncomine Dx Target Test	(%)	PNA‐LNA PCR clamp	(%)
Success of analysis	104	90%	116	100%
Not passing the nucleic acid concentration threshold	3	3%	0	0%
Invalid results for all *EGFR* mutations	7	6%	0	0%
Invalid results for subset of *EGFR* mutations	2	2%	0	0%

EGFR, epidermal growth factor receptor.

**Figure 1:**
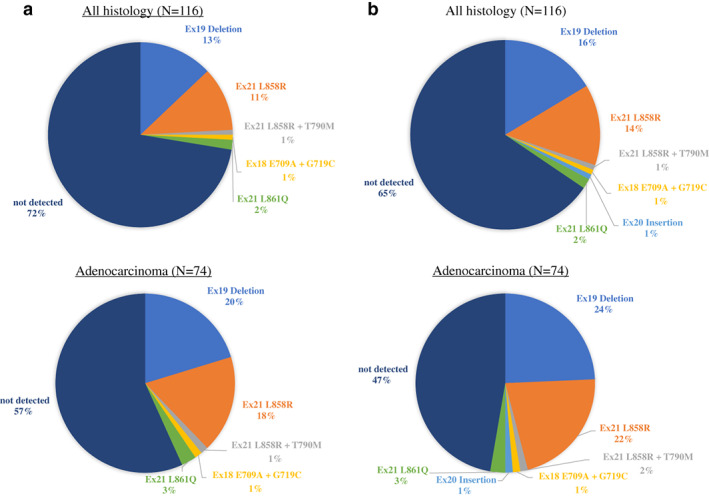
The detection rates for *EGFR* mutations. (**a**) Detection rate of the Oncomine Dx Target Test by all histology and adenocarcinoma. (**b**) Detection rate of the PNA‐LNA PCR clamp test by all histology and adenocarcinoma.

The detection rates for *EGFR* mutations of each test for all samples, and adenocarcinoma, are shown in Fig 1. Although the detection rate of the Oncomine Dx Target Test was 28%, the rate of the PNA‐LNA PCR clamp test was 35% for all samples (*P* = 0.32). For adenocarcinoma, the detection rates were 43% for the Oncomine Dx Target Test, and 53% for the PNA‐LNA PCR clamp test (*P* = 0.32). Among all samples, 36 common mutations and six uncommon mutations, including two compound mutations were detected by the PNA‐LNA PCR clamp test. Three samples with exon 21 p.L858R could not be detected by the Oncomine Dx Target Test due to a failure to pass the nucleic acid concentration threshold and invalid results. Among the samples successfully analyzed by each test, five discordant results were reported, and the concordance rate was 95%. The Oncomine Dx Target Test failed to detect four exon 19 deletion mutations that were detected with the PNA‐LNA PCR clamp test. Although the Oncomine Dx Target Test reported a negative for *EGFR* mutation in one sample where exon 20 insertion was detected by the PNA‐LNA PCR clamp test, the Oncomine Dx Target Test did not include exon 20 insertion as a target mutation to be reported when the sample was analyzed.

### Clinical information of discordant cases

The four discordant cases of exon 19 deletion between the Oncomine Dx Target Test and the PNA‐LNA PCR clamp test are detailed in Table 3. Although all samples were small biopsy samples collected by EBB/TBB, these had a sufficient amount of tumor cells and content to be viable. Tumor histology of three cases were adenocarcinoma, and one was large cell carcinoma (LCC). One patient had disease progression early after the administration of EGFR‐TKI, and another patient who was administered EGFR‐TKI could not have their response assessed due to an adverse event of pneumonitis. Two early stage patients underwent surgery and were not administered EGFR‐TKI.

**Table 3:** Clinical information of discordant cases

					Result of *EGFR* mutation				
No	Age	Sex	Sampling method	Histology	Oncomine Dx Target Test	PNA‐LNA PCR clamp	Stage (UICC‐eighth)	Treatment	Response to EGFR‐TKI
1	47	M	TBB	ADC	Negative	Detected exon 19 deletion	IVB	Osimertinib	PD
2	70	M	EBB	LCC	Negative	Detected exon 19 deletion	Postoperative recurrence	Osimertinib	NA due to pneumonitis
3	68	M	TBB	ADC	Negative	Detected exon 19 deletion	IA3	Operation	Not administered
4	68	F	TBB	ADC	Negative	Detected exon 19 deletion	IB	Operation	Not administered

ADC, adenocarcinoma; EBB, endobronchial biopsy; EGFR, epidermal growth factor receptor; LCC, large cell carcinoma; NA, not assessed; PD, progression disease; TBB, transbronchial biopsy; TKI, tyrosine kinase inhibitor; UICC, Union for International Cancer Control.

## Discussion

This is the first report to indicate that the Oncomine Dx Target Test may not be comparable to conventional single gene testing for the detection of *EGFR* mutations. Among patients harboring *EGFR* mutations detected by the PNA‐LNA PCR clamp test, the Oncomine Dx Target Test was unable to detect seven *EGFR* mutations (6%). All were common mutations, specifically; one harboring exon 21 p.L858R due to not passing the nucleic acid concentration threshold, two harboring exon 21 p.L858R due to invalid results, and four harboring exon 19 deletion due to false negative results. In the current Japanese medical insurance system, the samples which fail analysis by the Oncomine Dx Target Test due to not passing the nucleic acid concentration threshold can be submitted for conventional single gene tests; however, the samples which fail due to invalid results cannot be submitted for conventional single gene tests using medical insurance in clinical practice. Missing suitable patients responsive to EGFR‐TKIs should be avoided, especially in a high prevalence area for *EGFR* mutations.^4^

With regard to the cases with discordant results, excluding the sample where exon 20 insertion was detected by the PNA‐LNA PCR clamp test, all samples were collected by EBB/TBB and exon 19 deletion could not be detected by the Oncomine Dx Target Test in our study. Two factors were considered to cause the difference in results between the tests; one is the lower sensitivity of the Oncomine Dx Target Test compared with the PNA‐LNA PCR clamp test, and the other is the difference in the range of variants reported by each test. The estimated limit of detections (LODs) of the Oncomine Dx Target Test has been previously reported to be 6% allele frequency (AF) for *EGFR* exon 19 deletion.^10^ A clinical bridging study to establish the assurance of the Oncomine Dx Target Test compared with the therascreen *EGFR* assay, whose LODs were 1%–2% for *EGFR* common mutations, was performed by Thermo Fisher Scientific and showed good concordance.^10^ Meanwhile, the LODs for the PNA‐LNA PCR clamp test have been estimated to be 0.1%–1%^7^; therefore, it is expected that the PNA‐LNA PCR clamp test is able to detect a lower concentration of *EGFR* mutation. In cases 1 and 2, the Catalogue of Somatic Mutations in Cancer (COSMIC) IDs of the exon 19 deletions identified by the PNA‐LNA PCR clamp test, were variants that are detectable by the Oncomine Dx Taget Test. Therefore, the cause of the discordant results for these two cases would likely be the lower sensitivity of the Oncomine Dx Target Test. The COSMIC IDs in cases 3 and 4 could not be identified; therefore, the cause of the discordant results was unknown. When the results of the Oncomine Dx Target Test for samples with high tumor content were false negative due to low sensitivity, the clinical meaning of detecting these genetic abnormalities was unclear because the tumors harboring the mutation may not have been dominant in the whole tumor.^11^ Meanwhile, when the false negative results due to low sensitivity come from low tumor content, trimming or macro‐ and microdissection may be useful to eliminate nontumor cells, and EGFR‐TKI would be effective on the tumors. Although the sample in Case 1 had enough tumor content, Case 1 did not respond to EGFR‐TKI. We speculate that this was due to the sample being taken from a heterogeneous tumor, in which the areas that harbored the mutation did not form the dominant portion of the tumor. This resulted in the tumor sample failing to meet the sensitivity limit of the Oncomine Dx Target Test, the discordant results between the Oncomine Dx Target Test and the PNA‐LNA PCR clamp test, and the lack of response to EGFR‐TKI.

There were several limitations to this study. First, this was a relatively small retrospective study. Although the results of this study suggested that the Oncomine Dx Target Test had a risk of false negatives for *EGFR* mutations, especially exon 19 deletion variants, more samples would be needed to evaluate the frequency of false negatives for each variant and the clinical meaning of detecting the mutations. Second, this study was conducted in a single institute, and therefore the results of this study may not be applicable to other institutions because the methods of tissue sampling, sample preparation process, and judgment of whether or not to submit a sample for Oncomine Dx Target Test, vary in each institution. Finally, we did not perform macro‐ and microdissection; however, macro‐ and microdissection would further improve the tumor content and decrease the false negative rate.

In conclusion, the success rate of Oncomine Dx Target Test analysis is generally well tolerated; however, we should consider the risk of missing *EGFR* mutations compared with conventional single gene testing due to invalid and false‐negative results.

## Disclosure

Matsusaka Municipal Hospital received research grant funding from Novartis, GlaxoSmithKline, AstraZeneca, Daiichi Sankyo, Bayer, and Boehringer Ingelheim. K. Ito has received speaker fees as honoraria from Eli Lilly Japan, Chugai, AstraZeneca, MSD, Boehringer Ingelheim Japan, Ono, and Pfizer Japan. O. Taguchi received speaker fees as honoraria from AstraZeneca. O. Hataji received speaker fees as honoraria from Novartis Pharma, AstraZeneca, and Boehringer Ingelheim Japan. The remaining authors declare no conflict of interest.

## Supporting information


**Table S1.** List of genes searched for in the Oncomine Dx Target Test.
