# Supplementary material for: Comparison of the analytical performance between the Oncomine Dx Target Test and a conventional single gene test for epidermal growth factor receptor mutation in non‐small cell lung cancer
Source: Thorac Cancer. 2020 Dec 12;12(4):462–7. doi: 10.1111/1759-7714.13767 (PMC7882374; doi:10.1111/1759-7714.13767)
Supplement: Supplementary file 1 — Table S1. List of genes searched for in the Oncomine Dx Target Test. [file TCA-12-462-s001.docx]

**Supplemental Table 1.** **List of genes searched for in the Oncomine Dx Target Test**

| Gene Symbol | Approved name by HUGO Gene Nomenclature Committee (HGNC) |
| --- | --- |
| *ABL1* | ABL proto-oncogene 1, non-receptor tyrosine kinase |
| *AKT1* | AKT serine/threonine kinase 1 |
| *ALK* | ALK receptor tyrosine kinase |
| *AR* | androgen receptor |
| *AXL* | AXL receptor tyrosine kinase |
| *BRAF* | B-Raf proto-oncogene, serine/threonine kinase |
| *CDK4* | cyclin dependent kinase 4 |
| *CTNNB1* | catenin beta 1 |
| *DDR2* | discoidin domain receptor tyrosine kinase 2 |
| *EGFR* | epidermal growth factor receptor |
| *ERBB2* | erb-b2 receptor tyrosine kinase 2 |
| *ERBB3* | erb-b2 receptor tyrosine kinase 3 |
| *ERBB4* | erb-b2 receptor tyrosine kinase 4 |
| *ERG* | ETS transcription factor ERG |
| *ESR1* | estrogen receptor 1 |
| *ETV1* | ETS variant transcription factor 1 |
| *ETV4* | ETS variant transcription factor 4 |
| *ETV5* | ETS variant transcription factor 5 |
| *FGFR1* | fibroblast growth factor receptor 1 |
| *FGFR2* | fibroblast growth factor receptor 2 |
| *FGFR3* | fibroblast growth factor receptor 3 |
| *GNA11* | G protein subunit alpha 11 |
| *GNAQ* | G protein subunit alpha q |
| *HRAS* | HRas proto-oncogene, GTPase |
| *IDH1* | isocitrate dehydrogenase (NADP(+)) 1 |
| *IDH2* | isocitrate dehydrogenase (NADP(+)) 2 |
| *JAK1* | Janus kinase 1 |
| *JAK2* | Janus kinase 2 |
| *JAK3* | Janus kinase 3 |
| *KIT* | KIT proto-oncogene, receptor tyrosine kinase |
| *KRAS* | KRAS proto-oncogene, GTPase |
| *MAP2K1* | mitogen-activated protein kinase kinase 1 |
| *MAP2K2* | mitogen-activated protein kinase kinase 2 |
| *MET* | MET proto-oncogene, receptor tyrosine kinase |
| *MTOR* | mechanistic target of rapamycin kinase |
| *NRAS* | NRAS proto-oncogene, GTPase |
| *NTRK1* | neurotrophic receptor tyrosine kinase 1 |
| *NTRK2* | neurotrophic receptor tyrosine kinase 2 |
| *NTRK3* | neurotrophic receptor tyrosine kinase 3 |
| *PDGFRA* | platelet derived growth factor receptor alpha |
| *PIK3CA* | phosphatidylinositol-4,5-bisphosphate 3-kinase catalytic subunit alpha |
| *PPARG* | peroxisome proliferator activated receptor gamma |
| *RAF1* | Raf-1 proto-oncogene, serine/threonine kinase |
| *RET* | ret proto-oncogene |
| *ROS1* | ROS proto-oncogene 1, receptor tyrosine kinase |
| *SMO* | smoothened, frizzled class receptor |
